# The Endothelial Cell-Related Genes *EIF1* and *HSPA1B* Contribute to the Pathogenesis of Alzheimer’s Disease by Modulating Peripheral Immunoinflammatory Responses

**DOI:** 10.3390/brainsci15020205

**Published:** 2025-02-16

**Authors:** Yucheng Gu, Nihong Chen, Jingwen Qi, Lin Zhu, Xiangliang Chen, Feng Wang, Yingdong Zhang, Teng Jiang

**Affiliations:** 1 Department of Neurology, Nanjing First Hospital, Nanjing Medical University, No. 68 Changle Road, Nanjing 210006, China; seumedgyc@yeah.net (Y.G.); chennihong@njmu.edu.cn (N.C.); qijingwen@stu.njmu.edu.cn (J.Q.); zhulin1018@njmu.edu.cn (L.Z.); chenxl@njmu.edu.cn (X.C.); zhangyingdong@njmu.edu.cn (Y.Z.); 2 Department of Nuclear Medicine, Nanjing First Hospital, Nanjing Medical University, No. 68 Changle Road, Nanjing 210006, China; fengwangcn@njmu.edu.cn

**Keywords:** Alzheimer’s disease, single-cell RNA sequencing, endothelial cells, peripheral immunoinflammatory responses, *EIF1*, *HSPA1B*

## Abstract

Background: Emerging evidence suggests that peripheral immunoinflammatory responses contribute to Alzheimer’s disease (AD) pathogenesis, and endothelial cells (ECs) are involved in these responses. Nevertheless, the potential molecular mechanisms and signaling pathways by which ECs modulate peripheral immunoinflammatory responses and thus contribute to AD pathogenesis are not fully understood. Methods: The single-cell RNA sequencing dataset GSE157827 was analyzed, and AD key genes were screened using LASSO regression and random forest algorithms. Functional enrichment analyses of these AD key genes were conducted using gene set enrichment analysis (GSEA) and gene set variation analysis. Immune cell infiltration analyses for AD key genes were performed using single-sample GSEA, and their correlations with immunoinflammatory factors were assessed using the TISIDB database. Peripheral blood RNA sequencing data from our cohort were utilized to validate the expression patterns of EC-related AD key genes in peripheral blood and to investigate their association with cognition. Results: ECs are the most significant contributors to AD among all brain cell subpopulations. For the first time, the EC-related genes *EIF1* and *HSPA1B* were identified as key genes associated with AD progression. These two EC-related key genes may participate in AD pathogenesis by modulating peripheral immunoinflammatory responses. The levels of *EIF1* and *HSPA1B* were significantly altered in the peripheral blood during AD progression, and *EIF1* levels correlated with cognitive functions in AD clinical continuum patients. Conclusions: These findings underscore the critical roles of the EC-related genes *EIF1* and *HSPA1B* in AD pathogenesis and their potential as biomarkers for this disease.

## 1. Introduction

Alzheimer’s disease (AD) is a complex and progressively debilitating neurological disorder [1]. According to the World Health Organization, 44 million people worldwide suffer from AD. This number is expected to reach around 78 million in 2030 and 139 million in 2050 [2]. Its neuropathological hallmarks encompass the accumulation of amyloid-beta (Aβ), which develops into fibrillar plaques, the formation of neurofibrillary tangles from the hyperphosphorylated tau protein, neuroinflammation, and the degeneration of neurons and synapses [3]. To date, the pathogenesis of AD remains unclear. Meanwhile, there is an urgent need for reliable blood biomarkers to identify individuals at early stages for timely intervention.

It is well established that central neuroinflammation, driven by microglia and astrocytes, plays a crucial role in the progression of AD [4,5,6]. Emerging evidence suggests that peripheral immunoinflammatory responses also contribute to the pathogenesis of AD [7]. Blood from AD patients has shown significantly elevated levels of immunoinflammatory cytokines and a higher neutrophil-to-lymphocyte ratio, an indicator of peripheral immunoinflammatory responses, compared to normal control (NC) subjects [8]. Recent findings suggest that endothelial cells (ECs) participate in these peripheral immunoinflammatory responses [9]. They can directly induce immunoinflammatory actions by releasing proinflammatory cytokines and reactive oxygen species or regulate immunoinflammatory levels through immune-related receptors expressed on their surface [10]. However, the potential molecular mechanisms and signaling pathways by which ECs modulate peripheral immunoinflammatory responses and thus contribute to AD pathogenesis remain elusive.

Recent advances in single-cell RNA sequencing (scRNA-seq) have unveiled cell type-specific molecular alterations and significant heterogeneity within neurons, microglia, astrocytes, oligodendrocytes, and ECs in the brains of AD patients [11]. In this study, we examined the single-cell transcriptomic dataset GSE157827 [12,13], which includes prefrontal cortical samples from 12 AD patients and 9 NC subjects. Our analysis revealed that ECs are the most substantial contributors to AD among all brain cell subpopulations. Specifically, we identified the EC-related genes *EIF1* and *HSPA1B* as key genes associated with AD progression. These two EC-related genes may be involved in the pathogenesis of AD by influencing peripheral immunoinflammatory responses. Importantly, utilizing our own dataset containing 7 NC subjects and 23 AD clinical continuum patients [14], we demonstrated that the levels of *EIF1* and *HSPA1B* are significantly altered in the peripheral blood during AD progression. Furthermore, *EIF1* levels were found to correlate with cognitive functions in AD clinical continuum patients. These results highlight the critical roles of EC-related *EIF1* and *HSPA1B* in AD pathogenesis and suggest their potential as biomarkers for this disease.

## 2. Materials and Methods

### 2.1. Data Acquisition

The Gene Expression Omnibus (GEO) database (https://www.ncbi.nlm.nih.gov/geo/, accessed on 11 September 2024) is a repository for gene expression data, curated and sustained by the National Center for Biotechnology Information (NCBI). We retrieved the data file for GSE157827 (containing 12 AD patients and 9 NC subjects) from the NCBI GEO public database and extracted the single-cell expression profiles for further analysis [12,13]. Concurrently, our clinical dataset (PRJNA1064226) encompassed expression profiles from 30 subjects, comprising 7 NC subjects and 23 AD clinical continuum patients (mild cognitive impairment (MCI) patients: n = 8; patients with mild AD-related dementia (ADD): n = 7; patients with moderate ADD: n = 8; classified based on the global clinical dementia rating (CDR) scale). Detailed information regarding our clinical dataset is presented in Supplementary Table S1. The clinical data collection in this study was approved by the Ethics Committee of Nanjing First Hospital. Informed consent was obtained from all subjects involved in the study or their legal guardians.

### 2.2. Single-Cell Sequencing Analysis

Initially, the expression dataset was imported using the R package “Seurat” (10.32614/CRAN.package.Seurat, version 5.2.1), and genes with low expression levels were filtered out. Following this, the data underwent standardization, normalization, and principal component analysis (PCA). The optimal number of principal components was ascertained using an ElbowPlot, and the inter-cluster relationships were visualized through uniform manifold approximation and projection (UMAP) analysis. Clusters were annotated using marker genes, and cells potentially pivotal to disease onset were identified. Lastly, the FindMarkers function was utilized to identify marker genes for each cell subtype within the single-cell expression profile.

### 2.3. Contribution of Each Cell Subpopulation to AD

The contributions of various cell subpopulations to AD were assessed by examining alterations in cell count and gene expression profiles. Subsequently, we introduced the fold change score (*FCscore*), a metric that captures changes in both the quantity and expression levels of signature genes involved in biological processes [15]. This methodology entailed pinpointing the 100 most highly expressed genes in the control group compared to the disease group, which were then designated as characteristic genes for each group. Ultimately, the AD contribution of distinct cell types and subsets was delineated by the average *FCscore* of all characterized genes within this cluster.

### 2.4. Functional Analysis of Significant Genes

The R package “ClusterProfiler” (10.18129/B9.bioc.clusterProfiler, version 4.14.4) was employed to functionally annotate key genes, thereby enabling a comprehensive exploration of their functional correlations. Gene Ontology (GO) and the Kyoto Encyclopedia of Genes and Genomes (KEGG) were utilized to assess pertinent functional categories. Pathways enriched in both GO and the KEGG with *p*-values and q-values below 0.05 were deemed significant.

### 2.5. Identifying Key Genes for AD by Machine Learning Algorithms

We utilized Lasso logistic regression and random forest algorithms for feature selection to pinpoint key diagnostic genes associated with AD. The Lasso algorithm was executed via the R package “glmnet” (10.32614/CRAN.package.glmnet, version 4.1-8), while the random forest algorithm was leveraged to assess feature importance based on %IncMSE, ultimately selecting the top 10 features for further analysis.

### 2.6. Transcriptional Regulation Analysis of AD Key Genes

This study employed the R package “RcisTarget” (10.18129/B9.bioc.RcisTarget, version 1.26.0) to predict transcription factors, with all computations grounded in motif analysis. Beyond the motifs annotated by the source data, supplementary annotation files were derived using motif similarity and gene sequence information. The normalized enrichment score for each motif was computed from the area under the curve distribution of all motifs within the gene set.

### 2.7. Enrichment Analysis of AD Key Gene-Related Pathways

Gene set enrichment analysis (GSEA) was employed to further investigate the disparities in signaling pathways between groups exhibiting high and low expression levels. The reference gene set utilized was version 7.0, sourced from the Molecular Signatures Database (https://www.gsea-msigdb.org/gsea/msigdb, accessed on 22 October 2024) [16,17]. As a curated gene set specific to subtype pathways, differential expression analysis was conducted to assess pathway differences between subtypes. Significantly enriched gene sets were identified based on a concordance score (adjusted *p* < 0.05) and ranked accordingly. Subsequently, gene sets were downloaded from the Molecular Signatures Database and subjected to gene set variation analysis (GSVA) to comprehensively score each gene set, thereby evaluating potential alterations in biological functions across various samples.

### 2.8. Immune Cell Infiltration and Correlation Analysis

This study utilized the single-sample GSEA algorithm to quantify immune cell populations within the expression profiles of self-collected data, thereby inferring the relative proportions of 29 distinct types of immune-infiltrating cells [18]. We examined variations in immune cell infiltration between the disease and control cohorts and evaluated the associations between gene expression levels and immune cell content. Furthermore, we derived correlations between key genes and various immune factors from the TISIDB database (http://cis.hku.hk/TISIDB/, accessed on 22 October 2024) [19].

### 2.9. Gene Expression and Cognitive Correlation Analysis Across AD Continuum

A one-way analysis of variance was conducted to assess the expression differences in key genes among the NC group, MCI group, and ADD group, utilizing our proprietary clinical dataset (PRJNA1064226). Spearman correlation analysis was performed to uncover potential relationships between the expression levels of these key genes in the peripheral blood of individuals with normal cognitive function and AD clinical continuum patients. Details regarding the mini-mental state examination (MMSE), Montreal cognitive assessment (MoCA), and CDR-sum of boxes (CDR-SB) in AD clinical continuum patients are provided in Supplementary Table S1.

### 2.10. Statistical Analysis

All statistical analyses were performed using R software (version 4.2.2), and *p* < 0.05 was considered statistically significant.

## 3. Results

### 3.1. ECs Are the Most Significant Contributors to AD Among All Cell Subpopulations in the Brain

**Figure 1:**
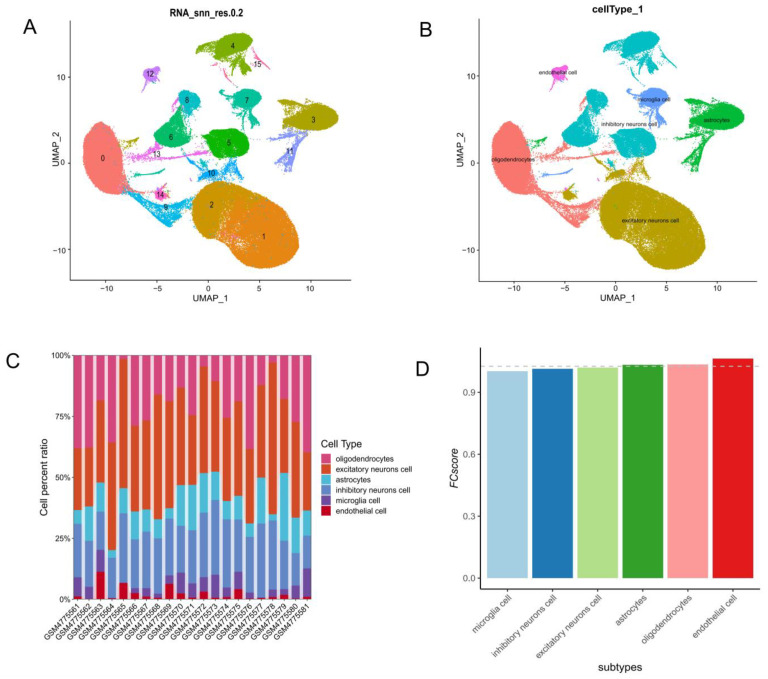
ECs are the most significant contributors to AD among all cell subpopulations in the brain. (**A**) UMAP plot of all cells in the GSE157827 dataset, highlighting clustering into 16 subtypes based on transcriptomic profiles, with numeric labels in various colors representing individual clusters. (**B**) UMAP plot annotated with six major cell types: oligodendrocytes, excitatory neurons, astrocytes, inhibitory neurons, microglia, and ECs. The spatial distribution highlights the clustering of distinct cell populations, reflecting their transcriptional heterogeneity. (**C**) Stacked bar plot displaying the percentage composition of each cell type across samples, revealing variability in cell type proportions among individuals. (**D**) Bar plot of fold change scores (*FCscore*), quantifying the contribution of each major cell type to AD.

In this study, we utilized the GSE157827 dataset, containing prefrontal cortical samples from 12 AD patients and 9 NC subjects [12,13]. The expression profile was processed through the Seurat package, and low-expression genes were filtered out based on violin plots and scatter plots (nFeature_RNA > 500, nCount_RNA > 1000, percent.mt < 20, nFeature_RNA < 6000, and nCount_RNA < 20,000). A total of 141,213 cells were retained. The gene expression profiles, with an emphasis on high-variance genes, along with details on normalization, PCA, and Harmony analysis, are shown in Supplementary Figure S1. Through UMAP analysis, we identified 16 cell subtypes (Figure 1A), which were further annotated and categorized into six major types: oligodendrocytes, excitatory neurons, astrocytes, inhibitory neurons, microglia, and ECs (Figure 1B). As shown in Figure 1C, there were distinct differences in the proportions of cell types across samples, with oligodendrocytes and excitatory neurons being the most prevalent, while ECs were the least frequent. After screening for the top 100 highly differentially expressed genes between the AD and NC groups from the GSE157827 dataset and calculating the *FCscore*, we found that ECs contribute most significantly to AD (Figure 1D). Consequently, we selected EC marker genes as the candidate gene set, applying filtering conditions of logFC > 1 and *P*_val_adj < 0.05, resulting in the inclusion of 432 genes in total. The results of GO and KEGG enrichment analyses for these EC marker genes are presented in Supplementary Figure S2A,B.

### 3.2. Identification of EC-Related EIF1 and HSPA1B as Key Genes in AD Progression

**Figure 2:**
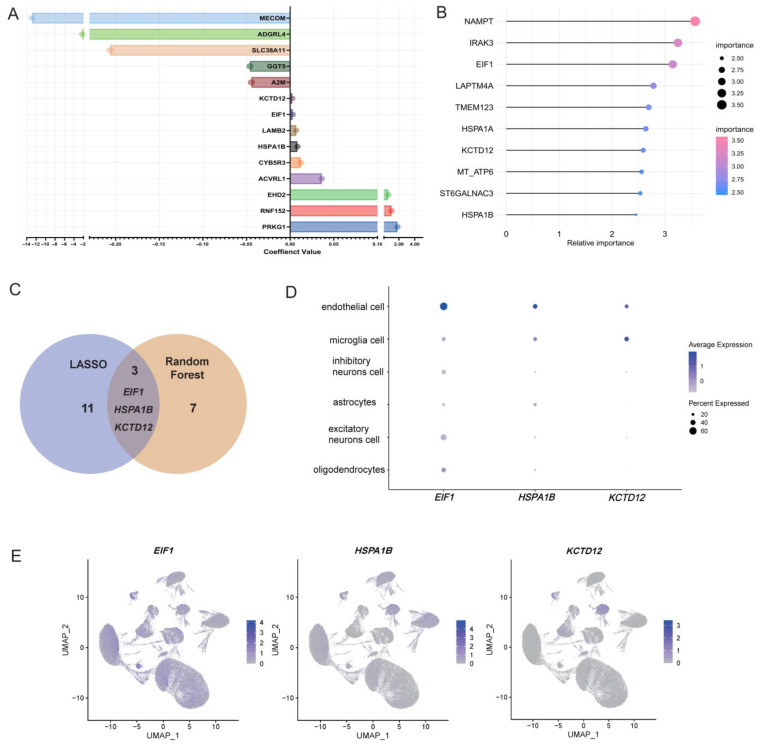
Identification of EC−related key genes for AD through machine learning. (**A**) Coefficient values of the 14 characteristic genes of AD identified by LASSO regression. (**B**) Relative importance of top 10-ranking genes identified by the random forest algorithm. (**C**) Venn diagram displaying the overlap of three key genes (*EIF1*, *HSPA1B*, and *KCTD12*) identified by LASSO regression and random forest analysis. (**D**) Dot plot showing the expression levels of *EIF1*, *HSPA1B*, and *KCTD12* across six major cell types. (**E**) UMAP plots illustrating the percentage of cells expressing the genes *EIF1*, *HSPA1B*, and *KCTD12* within different cell types, respectively. It should be noted that *KCTD12* exhibited its highest expression in microglial cells rather than ECs. Consequently, *EIF1* and *HSPA1B* were selected for further investigation in the present study as pivotal genes associated with AD.

To identify key genes associated with AD progression, EC marker genes identified in the previous step were used to screen characteristic genes through LASSO regression and random forest analysis. Lasso regression identified 14 genes as characteristic genes of AD (Figure 2A and Supplementary Figure S3A,B), and the top 10-ranked AD characteristic genes identified by the random forest algorithm are shown in Figure 2B and Supplementary Figure S3C. Interestingly, these two analyses revealed a convergence of three genes, namely *EIF1*, *HSPA1B* and *KCTD12* (Figure 2C). The expression profiles of these three genes were subsequently examined at the single-cell level and visualized across various cell types, including oligodendrocytes, excitatory neurons, astrocytes, inhibitory neurons, microglia, and ECs (Figure 2D,E). It should be noted that *KCTD12* exhibited its highest expression in microglial cells rather than ECs. Consequently, *EIF1* and *HSPA1B* were selected for further investigation in the present study as pivotal genes associated with AD.

### 3.3. Transcriptional Regulation and Enriched Signaling Pathways of EIF1 and HSPA1B in AD

**Figure 3:**
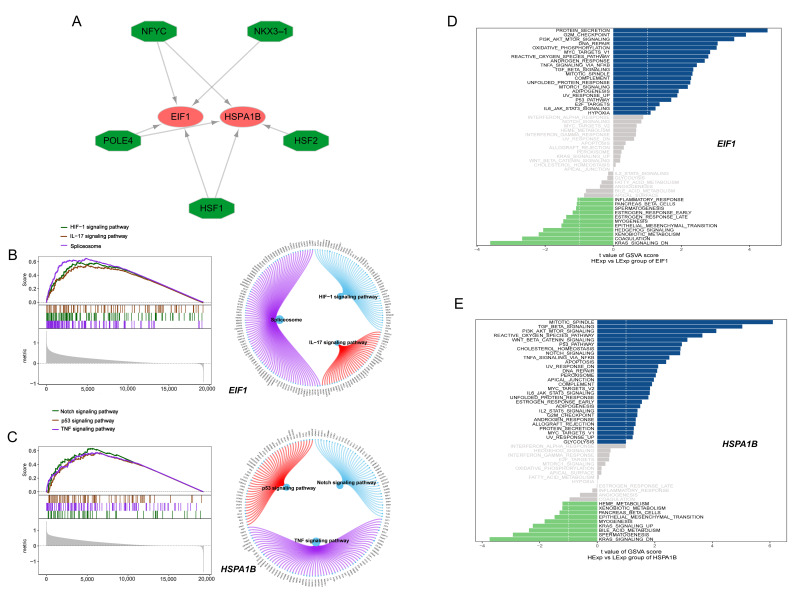
Transcriptional regulation and pathway enrichment of EC−related key genes in AD. (**A**) Predicted motifs associated with the regulation of *EIF1* and *HSPA1B* based on motif enrichment analysis. (**B**) Gene set enrichment analysis (GSEA) indicates that *EIF1* is associated with the HIF-1 signaling pathway, IL-17 signaling pathway, and spliceosome. (**C**) GSEA indicates that *HSPA1B* is linked to the Notch signaling pathway, p53 signaling pathway, and TNF signaling pathway. (**D**) Gene set variation analysis (GSVA) of pathways associated with *EIF1*. GSVA scores are compared for high versus low expression groups of *EIF1*. High expression of *EIF1* correlates with pathways including DNA repair, PI3K-Akt-mTOR signaling, and protein secretion. (**E**) GSVA of pathways associated with *HSPA1B*. GSVA scores are compared for high versus low expression groups of *HSPA1B*. High expression of *HSPA1B* is associated with TGF-β signaling, PI3K-Akt-mTOR signaling, and the p53 pathway.

The subsequent analysis utilized *EIF1* and *HSPA1B* as the gene set, revealing shared regulatory mechanisms, including those mediated by multiple transcription factors. Cumulative recovery curves were employed for the enrichment analysis of these transcription factors. The motif-TF annotation and selection analysis identified cisbp__M1269 (NES: 8.67) as the motif with the highest normalized enrichment score (Supplementary Figure S4). Enriched motifs and their corresponding transcription factors were visualized using Cytoscape, highlighting the participation of HSF1, HSF2, POLE4, NFYC, and NKC3-1 in the transcriptional network (Figure 3A). The GSEA results indicated that *EIF1* is associated with pathways such as the HIF-1 signaling pathway, IL-17 signaling pathway, and spliceosome (Figure 3B), whereas *HSPA1B* is linked to the Notch signaling pathway, p53 signaling pathway, and TNF signaling pathway (Figure 3C). The GSVA results suggested that a high expression of *EIF1* correlates with pathways including DNA repair, PI3K-Akt-mTOR signaling, and protein secretion (Figure 3D), while a high expression of *HSPA1B* is associated with TGF-β signaling, PI3K-Akt-mTOR signaling, and the p53 pathway (Figure 3E).

### 3.4. Immune Infiltration Analysis Highlights Interactions of EC-Related Genes EIF1 and HSPA1B with Peripheral Immunoinflammatory Processes in AD

**Figure 4:**
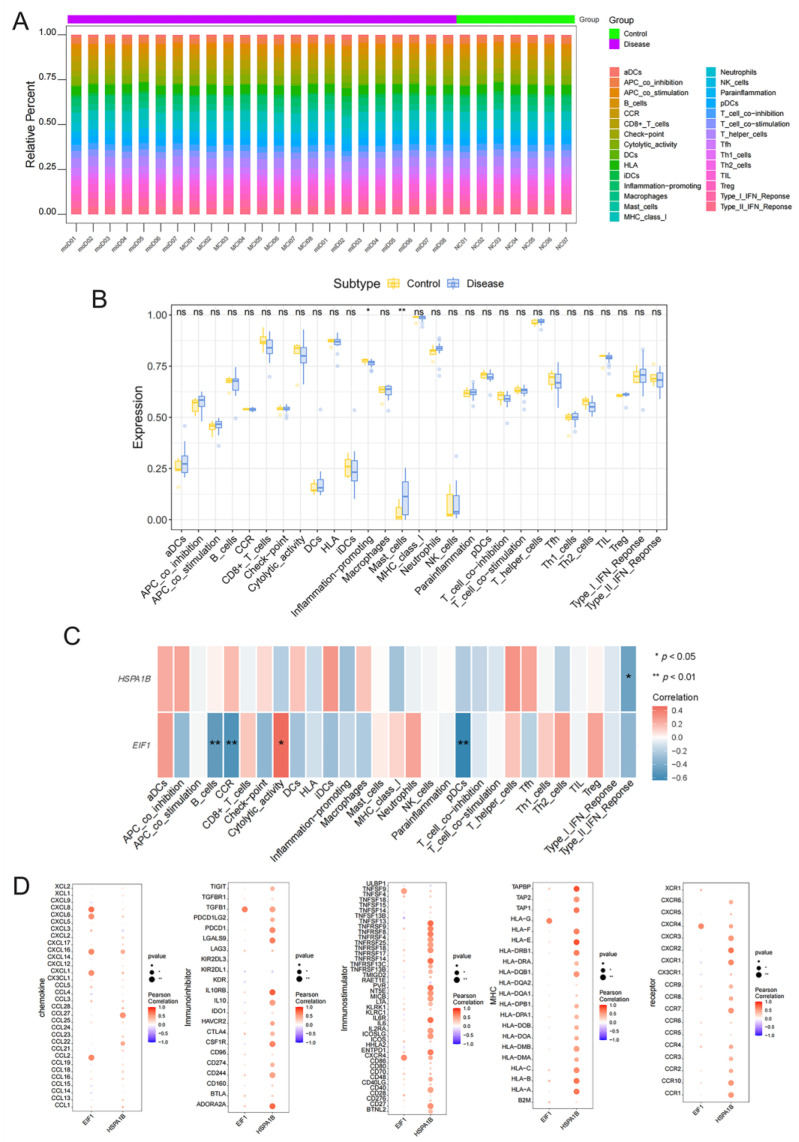
Immune infiltration analysis highlights the interactions of EC−related key genes with peripheral immunoinflammatory processes in AD. (**A**) Heatmap depicting the relative abundance of 29 immune cell types across different groups in our own dataset containing 7 NC subjects and 23 AD clinical continuum patients (8 patients with MCI, 7 patients with mild ADD, and 8 patients with moderate ADD). (**B**) Boxplot of immune cell infiltration scores, showing differences between NC subjects and AD clinical continuum patients. (**C**) Correlation between endothelial *EIF1* and *HSPA1B* expression and immune infiltration markers. (**D**) Dot plots showing the correlation of *EIF1* and *HSPA1B* with various immune factors, including chemokines, immunosuppressive factors, immunostimulatory factors, MHCs, and receptors (data downloaded from TISIDB database). * *p* < 0.05; ** *p* < 0.01, ns = not significant.

Increasing evidence indicates that peripheral immunoinflammatory processes play a crucial role in the progression of AD. To validate this notion, our own dataset was utilized. The distribution of peripheral immune cells in each patient is depicted in Figure 4A, and notable differences in the abundance of inflammation-promoting cells and mast cells were observed between AD clinical continuum patients and NC subjects (Figure 4B). Further investigation demonstrated that *EIF1* displayed a significant positive correlation with cytolytic activity and a significant negative correlation with B cells, CCR, pDCs, and other immune cells. *HSPA1B* was inversely correlated with the Type II IFN response (Figure 4C). Moreover, by utilizing the TISIDB database (http://cis.hku.hk/TISIDB/, accessed on 22 October 2024) [19], multiple significant correlations were observed between *EIF1* and *HSPA1B* with various immune factors, including chemokines, immunosuppressive factors, immunostimulatory factors, MHCs, and receptors (Figure 4D).

### 3.5. Dynamic Expression Patterns of EIF1 and HSPA1B in Peripheral Blood and Their Association with Cognitive Functions of AD Clinical Continuum Patients

**Figure 5:**
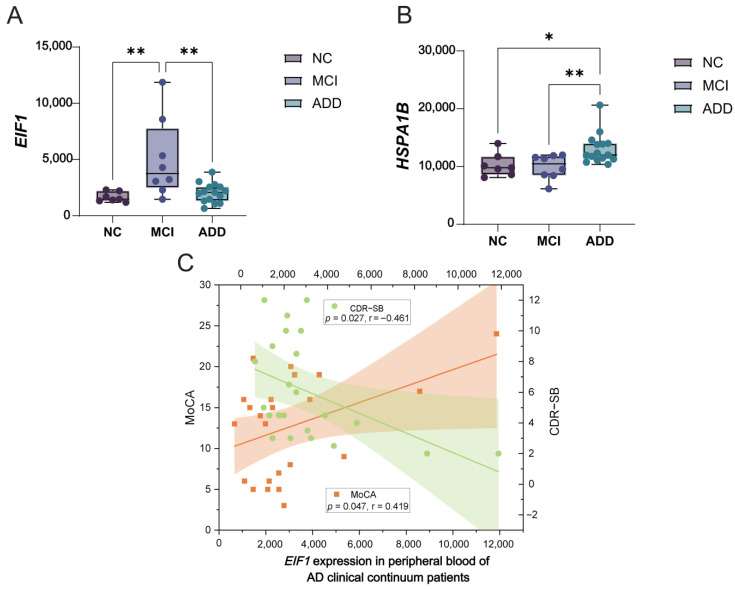
Dynamic expression patterns of *EIF1* and *HSPA1B* in peripheral blood and their association with cognitive functions of AD clinical continuum patients. (**A**) Boxplots displaying the dynamic alteration of *EIF1* in the peripheral blood across different AD stages. (**B**) Boxplots displaying the dynamic alteration of *HSPA1B* in the peripheral blood across different AD stages. (**C**) Scatter plot showing the correlation between *EIF1* expression and MoCA or CDR−SB scores in AD clinical continuum patients. * *p* < 0.05; ** *p* < 0.01.

First, to investigate the dynamic expression patterns of *EIF1* and *HSPA1B* in peripheral blood across different AD stages, our own dataset was used. As indicated by Figure 5A, *EIF1* expression was significantly upregulated in the MCI group and subsequently downregulated in the ADD patients. Meanwhile, *HSPA1B* expression was significantly higher in the ADD patients compared to the NC subjects or the MCI patients (Figure 5B). As shown by Figure 5C, correlation analysis for AD clinical continuum patients revealed that *EIF1* expression was positively correlated with MoCA scores (*p* = 0.047, *R* = 0.419) and negatively correlated with CDR-SB scores (*p* = 0.027, *R* = −0.461). No significant association was observed between *EIF1* expression and cognitive functions in the NC subjects. Additionally, *HSPA1B* expression did not correlate with cognitive functions in either the NC subjects or the AD clinical continuum patients.

## 4. Discussion

Brain ECs regulate the passage of substances into the brain and form the blood–brain barrier [20]. Increasing evidence suggests that ECs in the brain may be associated with the progression of AD. Previous GWASs have revealed that ECs in the brain microvasculature express the highest number of AD risk genes [21,22]. Furthermore, recent studies utilizing human brain samples have explored endothelial transcriptomic heterogeneity and BBB pathophysiology by comparing aged NC brains with those affected by AD, uncovering significant changes in endothelial gene expression patterns [21,22]. Additionally, the dysfunction of brain ECs is observed in the early stages of AD [23]. Here, we conducted an scRNA-seq analysis of the GSE157827 dataset, containing prefrontal cortical samples from 12 AD patients and 9 NC subjects [12,13]. In line with the above findings, our analysis demonstrated that ECs, despite their small proportion in the brain, exert the most significant effects on AD pathogenesis compared to other cell types.

Next, employing Lasso regression and random forest feature selection on the scRNA-seq data, we identified *EIF1* and *HSPA1B* as the AD key genes predominantly expressed by ECs in our study. *EIF1*, a eukaryotic initiation factor, is loaded onto mRNAs as part of the 43S initiation complex, playing a crucial role in start codon recognition [24]. To date, limited research has explored the connection between *EIF1* and AD. However, *EIF1* has been found in synapse-enriched data from the frontal lobe of AD patients [25], and its expression is revealed to increase in mature neurons within the aging brain [26]. In contrast, there has been a growing focus on the relationship between *HSPA1B* and AD in recent years. *HSPA1B*, a member of the heat shock protein 70 family, functions in conjunction with other heat shock proteins to stabilize existing proteins against aggregation and facilitates the folding of newly translated proteins in the cytosol and organelles [27]. Elevated levels of *HSPA1B* in human plasma have been linked to an increased long-term risk of AD [28]. Furthermore, polymorphisms in *HSPA1B* may be associated with behavioral and psychological symptoms in AD [29]. Additionally, the upregulation of *HSPA1B* has been observed in venule ECs in brain samples from AD patients [30], and it has been identified as a biomarker for programmed cell death in AD [31].

In addition to neuroinflammation in the brain, emerging evidence suggests that peripheral immunoinflammatory responses also participate in the pathogenesis of AD. Notably, blood from AD patients exhibited significantly elevated levels of immunoinflammatory cytokines, including IL-6, TNF-α, IL-1β, TGF-β, IL-12, and IL-18, compared to healthy controls [8]. Meanwhile, a higher neutrophil-to-lymphocyte ratio, an indicator of peripheral immunoinflammatory responses, was observed in the blood of AD patients compared to healthy subjects, correlating with more severe cognitive decline [32]. Consistent with these findings, we observed notable differences in the abundance of peripheral immune cells between AD clinical continuum patients and NC subjects in this study. It is important to note that ECs play a pivotal role in peripheral immunoinflammatory responses [33,34]. Upon stimulation, ECs can release proinflammatory cytokines and reactive oxygen species to directly induce immunoinflammatory actions [35]. Additionally, ECs can modulate immunoinflammatory levels through immune-related receptors expressed on their surface, such as the receptor for advanced glycation end products [36]. Interestingly, our GSEA revealed that the IL-17 pathway was involved in *EIF1*-mediated signaling, while the TNF pathway was involved in *HSPA1B*-related signaling. Since IL-17 and TNF are classic peripheral immunoinflammatory cytokines, our findings imply that these two AD key genes may be associated with immunoinflammatory activities induced by ECs. Furthermore, in the TISIDB database [19], significant correlations were observed between these two EC-related AD key genes and various immunoinflammatory factors, providing additional validation of our GSEA results. To our knowledge, this is the first study to report an association between the EC-related genes *EIF1* and *HSPA1B* and peripheral immunoinflammatory responses. In light of this evidence, we speculate that EC-related *EIF1* and *HSPA1B* may contribute to AD pathogenesis by modulating peripheral immunoinflammatory responses.

In this study, based on our dataset, we present initial evidence that the expression of *EIF1* and *HSPA1B* is significantly altered in the peripheral blood during AD progression. Specifically, *EIF1* expression was significantly upregulated in the MCI group and subsequently downregulated in the ADD patients, indicating that peripheral *EIF1* expression may serve as a biomarker for MCI. Conversely, *HSPA1B* expression was significantly higher in the ADD patients compared to the NC subjects or the MCI patients, suggesting that peripheral *HSPA1B* expression may predict disease progression from MCI to ADD. Additionally, we have demonstrated for the first time that peripheral *EIF1* levels correlate with cognitive functions in AD clinical continuum patients, further confirming the involvement of this EC-related gene in AD progression.

This study has several limitations. Firstly, the sample size for the peripheral blood transcriptomic analysis was relatively small, which may affect the robustness of our results. In the future, a larger cohort with more detailed neuropsychological data and comprehensive pathological diagnostic evidence may yield more compelling conclusions. Additionally, our study primarily relied on bioinformatics analyses and database mining. Do *EIF1* and *HSPA1B* have a direct causal relationship with immune cell abundance and immunoinflammatory responses? Do the expressions of *EIF1* and *HSPA1B* change in Aβ-stimulated ECs or ECs co-cultured with neutrophils? How do these two EC-related key genes regulate the IL-17 or TNF signaling pathways? These questions require further investigation using cellular and animal models of AD.

## 5. Conclusions

In summary, this study revealed that ECs are the most significant contributors to AD among all brain cell subpopulations. Specifically, the EC-related genes *EIF1* and *HSPA1B* were identified as key genes associated with AD progression. These two EC-related key genes may participate in AD pathogenesis by modulating peripheral immunoinflammatory responses. Additionally, the levels of *EIF1* and *HSPA1B* were significantly altered in the peripheral blood during AD progression, and *EIF1* levels correlated with cognitive functions in AD clinical continuum patients. These findings underscore the critical roles of the EC-related genes *EIF1* and *HSPA1B* in AD pathogenesis and their potential as biomarkers for this disease.

## Data Availability

The single-cell transcriptomic dataset GSE157827 is available in the NCBI GEO public database at https://www.ncbi.nlm.nih.gov/geo/query/acc.cgi?acc=GSE157827 (accessed on 11 September 2024). Meanwhile, our own clinical dataset PRJNA1064226 can be accessed in the NCBI BioProject database at https://www.ncbi.nlm.nih.gov/bioproject/PRJNA1064226 (accessed on 7 December 2024) upon request from the corresponding author.

## Supplementary Materials

The following supporting information can be downloaded at https://www.mdpi.com/article/10.3390/brainsci15020205/s1: Figure S1: Quality control and preprocessing of scRNA-seq data for analysis; Figure S2: Functional enrichment analysis of endothelial cell marker genes in AD; Figure S3: LASSO regression analysis and random forest analysis in AD key gene selection; Figure S4: The motif-TF annotation and selection analysis of AD key genes; Table S1: Demographics and neuropsychological test scores in our clinical dataset.
