# Supplementary material for: The Endothelial Cell-Related Genes EIF1 and HSPA1B Contribute to the Pathogenesis of Alzheimer’s Disease by Modulating Peripheral Immunoinflammatory Responses
Source: Brain Sci. 2025 Feb 16;15(2):205. doi: 10.3390/brainsci15020205 (PMC11852842; doi:10.3390/brainsci15020205)
Supplement: Supplementary file 1 [file brainsci-15-00205-s001.zip › Supplementary Figure Legends.pdf]

Supplementary Figure Legends

Supplementary Figure S1. Quality control and preprocessing of scRNA-seq data for analysis. (A) Violin plots displaying quality control metrics for each sample, including **nCount_RNA** (total number of RNA molecules), **nFeature_RNA** (number of genes detected per cell), and **percent.mt** (percentage of mitochondrial gene expression). **(B)** Scatter plots showing correlations between **nCount_RNA**, **nFeature_RNA**, and **percent.mt**. The positive correlation between **nCount_RNA** and **nFeature_RNA** indicates consistency in data quality, while **percent.mt** is negatively correlated, ensuring minimal mitochondrial contamination. **(C)** Variance plot showing the standardized variance against the average gene expression. Highly variable genes (**variable count: 2000**) were selected for downstream analysis to capture meaningful biological differences between cell types. **(D)** Principal Component Analysis (PCA) plot displaying the first two principal components, demonstrating clustering of cells based on major variance in the data. Cells are color-coded by their sample identity (GSM identifiers). **(E)** Standard deviation of the principal components, used to select significant components for further analysis. **(F)** Harmony plot for batch effect correction, showing cells after correction of batch effects.

Supplementary Figure S2. Functional enrichment analysis of endothelial cell marker genes in AD. (A) Gene Ontology (GO) enrichment analysis of endothelial cell marker genes. The analysis shows significant enrichment in biological processes (BP), cellular components (CC), and molecular functions (MF), such as wound healing, transport across the blood-brain barrier, focal adhesion, and structural constituents of ribosome. **(B)** Kyoto Encyclopedia of Genes and Genomes (KEGG) pathway enrichment analysis of endothelial cell marker genes. The enriched pathways include the PI3K-Akt signaling pathway, leukocyte transendothelial migration, ECM-receptor interaction, and focal adhesion.

Supplementary Figure S3. LASSO regression analysis and random forest analysis in AD key gene selection. (A) Ten-time cross-verification of adjusted parameter selection in the LASSO regression analysis, each curve corresponds to one gene. **(B)** LASSO coefficient analysis. The solid vertical lines represent the partial likelihood deviance standard error. The dotted vertical line was drawn to select the optimal λ value with the minimum mean-squared error, identifying 14 candidate genes. **(C)** Random forest error curve showing model performance according to the number of trees.

Supplementary Figure S4. The motif-TF annotation and selection analysis of AD key genes. Note that he motif with the highest normalized enrichment score (NES: 8.67) is cisbp__M1269.
