# Supplementary material for: The Endothelial Cell-Related Genes EIF1 and HSPA1B Contribute to the Pathogenesis of Alzheimer’s Disease by Modulating Peripheral Immunoinflammatory Responses
Source: Brain Sci. 2025 Feb 16;15(2):205. doi: 10.3390/brainsci15020205 (PMC11852842; doi:10.3390/brainsci15020205)
Supplement: Supplementary file 1 [file brainsci-15-00205-s001.zip › Supplementary Table S1.pdf]

Supplementary Table S1. Demographics and neuropsychological test scores in our clinical dataset.

	NC (*n* = 7)	MCI (*n* = 8)	ADD (*n* = 15)	*F or* χ^2	*P*
**Age (Mean ± SD)**	65.00±3.83	71.50±8.38	73.20±10.11	2.188	0.132*
**Gender (male%)**	3 (42.86)	3 (37.50)	5 (33.33)	0.190	0.910#
**Education (Mean ± SD)**	12.57±1.51	10.63±4.10	8.33±5.70	2.092	0.143*
**MMSE (Mean ± SD)**	28.43±0.98	24.00±3.85	14.87±6.31	20.510	<0.01*
**MoCA (Mean ± SD)**	25.00±1.63	18.00±4.50	9.87±4.90	31.653	<0.01*
**CDR-SB (Mean ± SD)**	——	2.88±0.69	7.73±2.85	22.140	<0.01*

NC = normal cognition. MCI = mild cognitive impairment. ADD = Alzheimer's Disease-related dementia. MMSE = Mini Mental State Examination. MoCA = Montreal cognitive assessment. CDR-SB = clinical dementia rating sum of boxes. SD = standard deviation.

* analyzed using one-way ANOVA

# analyzed using Chi-Squared Test
