# Supplementary figures and images for: The Endothelial Cell-Related Genes EIF1 and HSPA1B Contribute to the Pathogenesis of Alzheimer’s Disease by Modulating Peripheral Immunoinflammatory Responses
Source: Brain Sci. 2025 Feb 16;15(2):205. doi: 10.3390/brainsci15020205 (PMC11852842; doi:10.3390/brainsci15020205)

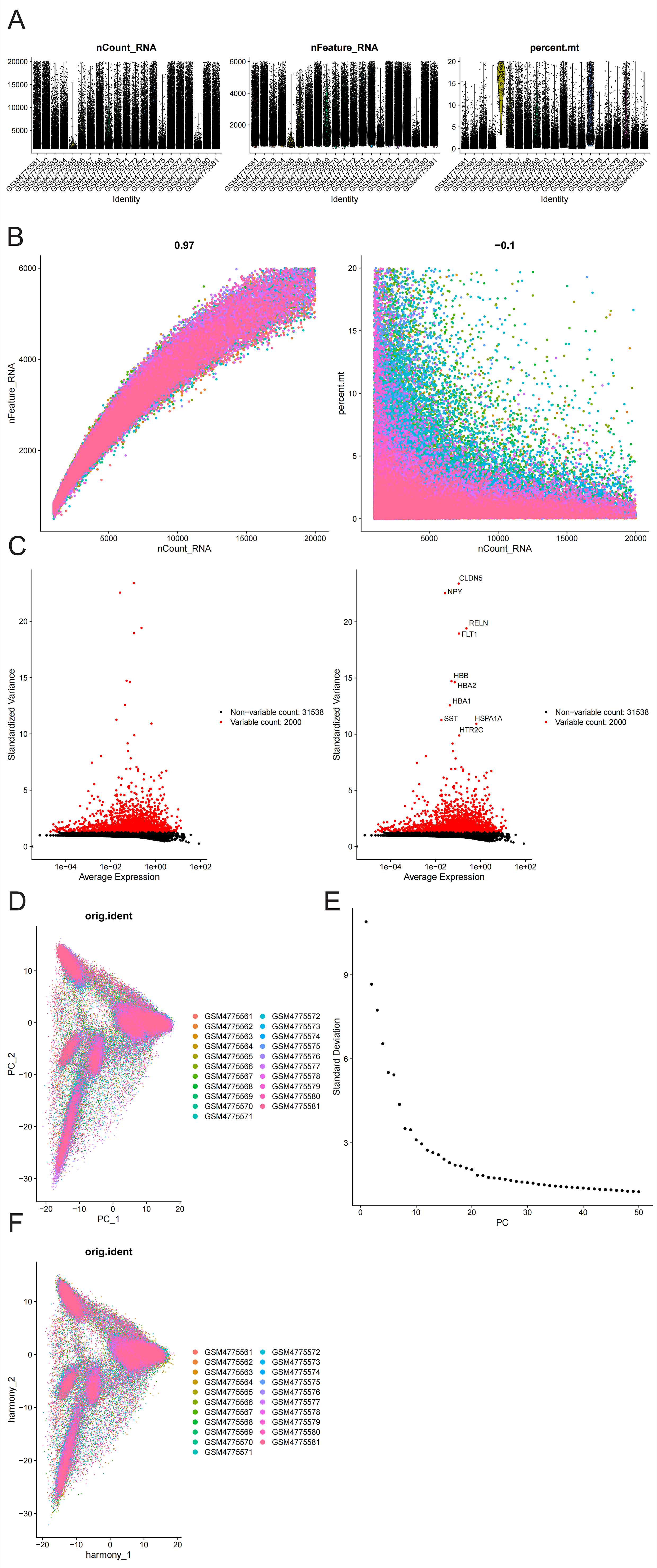

Supplement: Supplementary file 1 [file brainsci-15-00205-s001.zip › Supplementary Figure S1.tif]

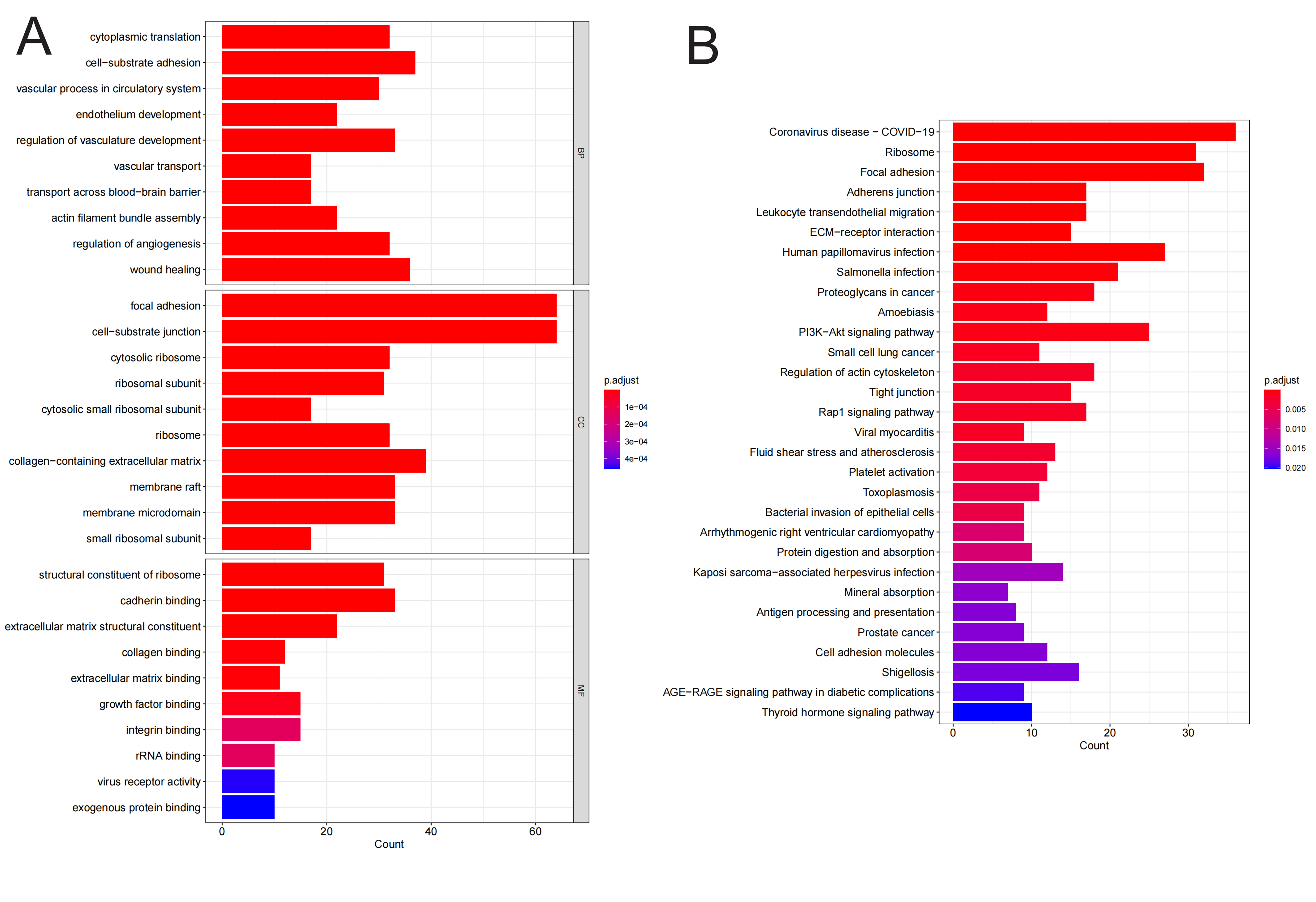

Supplement: Supplementary file 1 [file brainsci-15-00205-s001.zip › Supplementary Figure S2.tif]

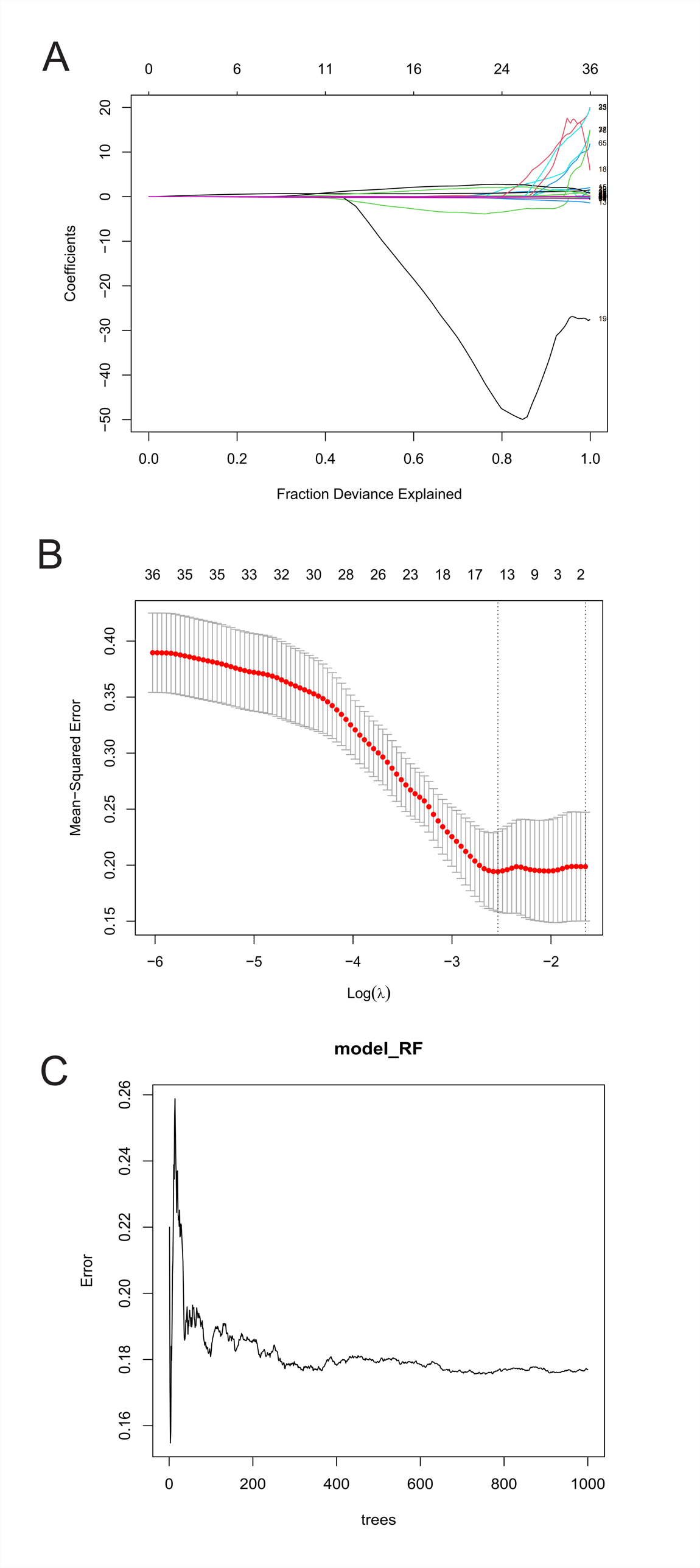

Supplement: Supplementary file 1 [file brainsci-15-00205-s001.zip › Supplementary Figure S3.tif]

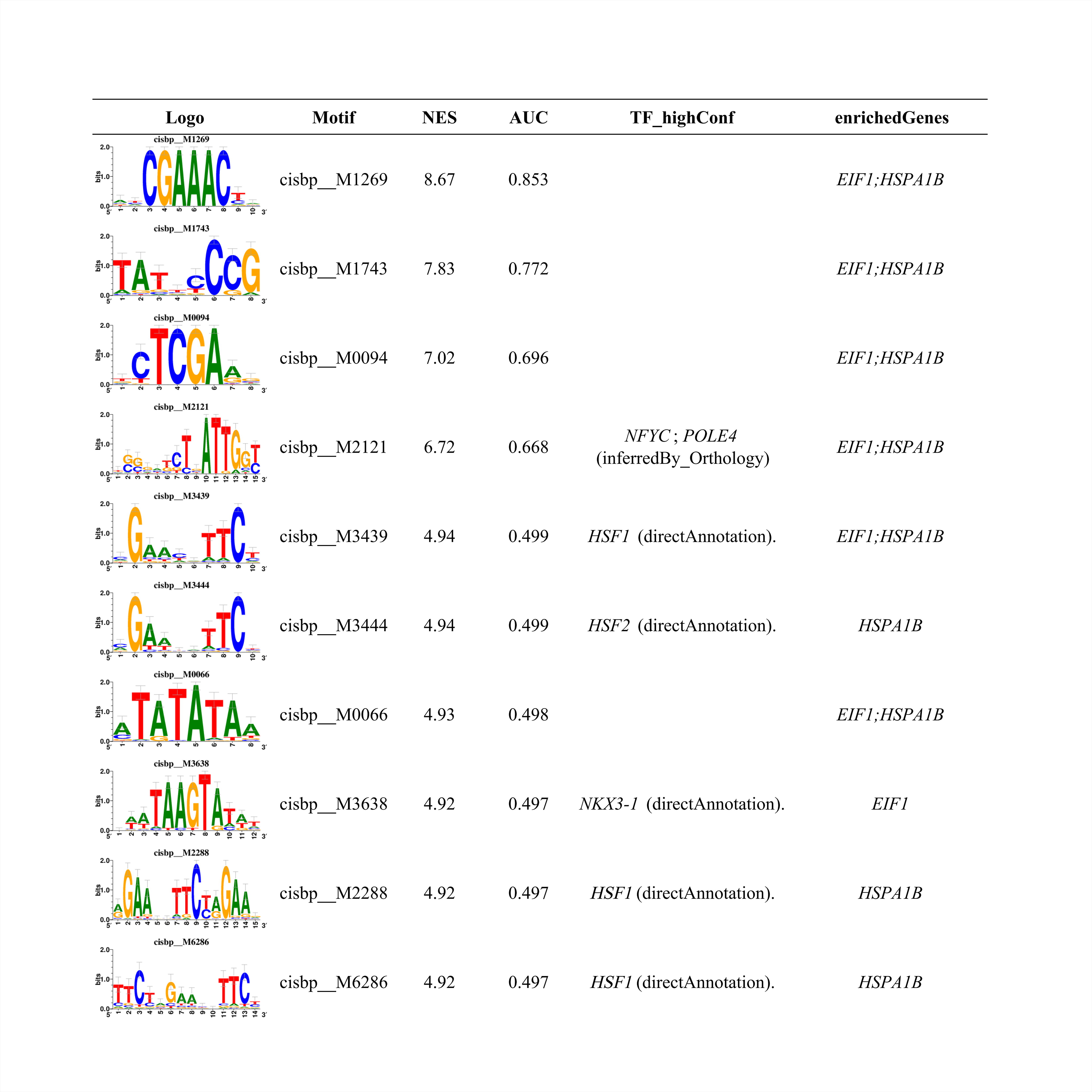

Supplement: Supplementary file 1 [file brainsci-15-00205-s001.zip › Supplementary Figure S4.tif]
